# Delayed Systemic Treatment with Cannabinoid Receptor 2 Agonist Mitigates Spinal Cord Injury-Induced Osteoporosis More Than Acute Treatment Directly after Injury

**DOI:** 10.1089/neur.2020.0059

**Published:** 2021-06-22

**Authors:** Michelle A. Tucci, Yilianys Pride, Suzanne Strickland, Susanna M. Salazar Marocho, Ramon J. Jackson, Joshua R. Jefferson, Alejandro R. Chade, Raymond J. Grill, Bernadette E. Grayson

**Affiliations:** ^1^ Department of Anesthesiology, University of Mississippi Medical Center, Jackson, Mississippi, USA.; ^2^ Department of Neurobiology and Anatomical Sciences, University of Mississippi Medical Center, Jackson, Mississippi, USA.; ^3^ Department of Biomedical Materials Science, University of Mississippi Medical Center, Jackson, Mississippi, USA.; ^4^ Department of Physiology and Biophysics, University of Mississippi Medical Center, Jackson, Mississippi, USA.; ^5^ Department of Medicine, University of Mississippi Medical Center, Jackson, Mississippi, USA.; ^6^ Department of Radiology, University of Mississippi Medical Center, Jackson, Mississippi, USA.

**Keywords:** cannabinoid receptor, osteoporosis, spinal cord injury

## Abstract

Nearly all persons with spinal cord injury (SCI) will develop osteoporosis following injury, and further, up to 50% of all persons with SCI will sustain a fracture during their lives. The unique mechanisms driving osteoporosis following SCI remain unknown. The cannabinoid system modulation of bone metabolism through cannabinoid 1/2 (CB1/2) has been of increasing interest for the preservation of bone mass and density in models of osteoporosis. Using a thoracic vertebral level 8 (T8) complete transection in a mouse model, we performed daily treatment with a selective CB2 receptor agonist, HU308, compared with SCI-vehicle-treated and naïve control animals either immediately after injury for 40 days, or in a delayed paradigm, following 3 months after injury. The goal was to prevent or potentially reverse SCI-induced osteoporosis. In the acute phase, administration of the CB2 agonist was not able to preserve the rapid loss of cancellous bone. In the delayed-treatment paradigm, in cortical bone, HU308 increased cortical-area to total-area ratio and periosteal perimeter in the femur, and improved bone density in the distal femur and proximal tibia. Further, we report changes to the metaphyseal periosteum with increased presence of adipocyte and fat mass in the periosteum of SCI animals, which was not present in naïve animals. The layer of fat increased markedly in HU308-treated animals compared with SCI-vehicle-treated animals. Overall, these data show that CB2 agonism targets a number of cell types that can influence overall bone quality.

## Introduction

Osteoporosis is a skeletal disease resulting in a reduction of bone mineral density (BMD) and bone mass with disorganization of trabecular bone architecture, leading to an increased incidence of bone fracture. The risk of osteoporosis is significantly greater in persons with spinal cord injury (SCI)-related paralysis than in age-matched uninjured individuals.^1,2^ Almost all patients with SCI will develop osteoporosis following injury.^3^ Further, nearly 50% of all persons with SCI will sustain a fracture during the course of their lives.^1^ Effective prevention of bone loss would improve the quality of life in persons with SCI.

The mechanisms driving osteoporosis following SCI are significantly different from age-related or post-menopausal osteoporosis.^4^ SCI-induced osteoporosis is caused by limb unloading due to injury coupled with neural denervation and vascular dysregulation within the bone; these processes are exacerbated in part by the inflammatory and endocrine changes that occur following SCI. Given that the bone mass mineral density of patients with SCI diminishes by 30% over the first 16–18 months after injury and reaches a steady-state within 2–5 years,^5^ early therapeutic interventions following SCI should have the potential for maximal bone preservation.

Chronic bone loss after SCI leads to an increased risk of low-impact fragility fractures,^6^ with the most common fracture sites located in the proximal tibia or distal femur.^7,8^ A number of factors influence bone mass in patients with SCI and include the level of the cord lesion, the extent of functional impairment, and the degree of muscular unloading of the bones. Bone loss is more pronounced in patients with complete than with incomplete SCI, and the risk of fracture increases with time and age post-injury.^7,9^ Therefore, certain subsets of individuals with SCI and osteoporosis are at greater risk for traumatic bone damage.

Pharmacological interventions for SCI-induced osteoporosis have focused on reversing bone resorption in the acute and rapid phases of bone loss.^10^ Bisphosphonates are the most studied pharmacological agents in the treatment of SCI-induced osteoporosis because they strongly inhibit osteoclastic bone resorption,^9^ and various studies have supported the use of the bisphosphonate class of drugs such as alendronate^11^ and zoledronic acid^12^ in the acute phase. Although bisphosphonates commonly are used for the long-term treatment of age/menopausal-induced osteoporosis, this class of drugs has poor patient adherence because of significant adverse side effects, including the development of kidney stones.^6^ Further, systematic reviews of bisphosphonate use in acute and chronic SCI concluded only mild attenuation of bone density loss with acute administration, which was not maintained during follow-up.^13,14^ In addition, Carbone and colleagues did not find a significant association between bisphosphonate use and incidence of lower-extremity fractures in men or women.^15^ A report by Anderson and Park concluded that there is no clear guidance on prophylactic intervention to prevent osteoporosis or stratify persons with SCI to their risk for fragility fractures in light that there is risk associated with using bisphosphonates in this vulnerable population.^16^ Thus, innovative drug targets and rehabilitative strategies are necessary for SCI-induced osteoporosis.

Members of the cannabinoid drug class recently have been shown to regulate bone growth under both normal and pathological conditions.^17–19^ The cannabinoid 1 (CB1) receptor is localized mainly to the brain and is responsible for the euphoria resulting from exposure to cannabinoids. The CB2 receptor, however, is localized throughout the periphery and is not associated with euphoria-like sensations. Stimulation of CB2 receptors, instead, produces potent anti-inflammatory effects, making CB2 receptor agonists a promising drug class for the development of novel therapeutics. The role of the endocannabinoid system in bone originally was elucidated using mouse models in which CB1 (*cnr1*) and CB2 (*cnr2*) were deleted.^17,18,20^ Deletion of *cnr1* led to overall increased bone mass, whereas *cnr2* deletion resulted in reduced bone mass similar to an osteoporosis-like phenotype in transgenic mice.^17,20^ Further, inactivation of CB1 has been shown to inhibit osteoclast activity and improve bone density, whereas CB2 agonism promotes bone growth through increasing osteoblast and inhibiting osteoclast activity.^19^ Finally, in a rodent model of menopause-induced osteoporosis, CB2 agonism attenuated ovariectomy-induced bone loss by markedly stimulating cortical thickness through suppression of osteoclast number and stimulation of bone formation.^19^

The CB2-selective agonists SR144528 and AM630 significantly inhibited osteoclast formation in bone marrow culture in a concentration-dependent fashion,^21^ further supporting a role for CB2 in both bone formation and bone resorption.^17,21^

In the current study, the goals were to determine whether a selective CB2 agonist could be used either to prevent the development of osteoporosis when delivered in acute SCI or to reverse it when treatment is delayed until a chronic time period in which osteoporosis has already developed. Specifically, we tested whether early, daily treatment with HU308, an agonist of the CB2 receptor, can prevent the onset of osteoporosis in mice following a full spinal transection injury in comparison with vehicle-treated injured mice and naïve controls. We further tested whether delaying treatment 3 months post-injury will reverse osteoporosis.

## Methods

### Animals

All procedures for animal use complied with the Guidelines for the Care and Use of Laboratory Animals by the National Research Council of the National Academies. Procedures were reviewed and approved by the University of Mississippi Medical Center (UMMC) Institutional Animal Care and Use Committee, #1482.

### Lesion model

Adult, male, C57BL/6N mice (age 6–7 weeks, 19–21 g; Harlan Laboratories, Indianapolis, IN, USA) were housed in groups upon arrival and maintained in the vivarium on a 12/12-h light/dark cycle at 25 °C and 50–60% humidity with *ad libitum* access to water. Animals were maintained on standard chow (#8640, Envigo; 3.0 kCal/g, 17% fat, 54% carbohydrate, 29% protein). Mice either received a full spinal transection lesion at thoracic vertebral level 8 (T8) or remained naïve to the procedure. Mice were anesthetized using a cocktail of ketamine (200 mg/kg) and xylazine (10 mg/kg). A laminectomy was performed at T8 under sterile surgical conditions. The spinal cord was completely severed at T8 using a combination of #11 scalpel blade and microscissors. Completeness of the lesion was confirmed visually by gently elevating the rostral and caudal poles of the lesion site. Following transection, a small amount of Gelfoam soaked in sterile 0.9% saline was placed over the injury site to enhance hemostasis. Muscles were sutured with 6-0 proline, and the skin of the back was closed with sterile, surgical staples.

Naïve, uninjured, age-matched mice served as controls for normal age-dependent alterations in bone density. Injured mice were also treated post-operatively with buprenorphine SR (1.2 mg/kg) to reduce pain and the antibiotic Baytril (5 mg/kg), to reduce risk of post-operative infection (over a period of 10 days, and then again as needed). Following surgery, injured subjects received manual bladder expression 3 times daily. Following surgical recovery, animals returned to multiple housing.

### Drug preparation/dosing regimen

The selective CB2 receptor agonist, HU308 (Tocris Biosciences, Minneapolis, MN, USA) was prepared daily in vehicle (VEH) containing ethanol, emulphor, and saline at a ratio of 1:1:18 and delivered via intraperitoneal (IP) injection at a concentration of 10 mg/kg (in a volume of 0.5 cc once daily for the stated duration of the experiment).

### Acute paradigm

Three hours post-SCI, injured mice were treated with HU308 (10 mg/kg) or vehicle via IP injection. Subjects were injected once daily for 40 days. The naïve group did not receive any injections. Final group sizes were as follows: naïve (*n* = 9), SCI-VEH (*n* = 9), and SCI-HU308 (*n* = 8).

### Delayed paradigm

In the delayed paradigm, animals received either surgery or were naïve to surgery, and then were allowed to recover for 3 months to produce the onset of osteoporosis.^22^ At this point, animals were injected daily with either VEH or HU308 for 30 days and then were euthanized; naive animals received no injection. Final animal numbers are as follows: naïve (*n* = 11), SCI-VEH (*n* = 12), and SCI- HU308 (*n* = 12).

### Micro-CT

Hind limbs were removed and frozen until scanning, which was performed using microtomography (Micro-CT; SkyScan 1076 system, Bruker BioSpin Corp., MA, USA). The reconstructed scans provided a three-dimensional (3D) rendered view of femurs and tibiae (right and left) in which trabecular bone density was assessed in the proximal and the distal region of the femurs and tibiae. Computed tomography (CT) software (CT-Analyser, Bruker Micro-CT, Kontich, Belgium) was used to calculate trabecular BMD. The cortical bone was analyzed 4 mm cranial from the knee growth plate for the femurs and 4 mm caudal from the knee growth plate for the tibiae, from which a volume of interest (VOI) 1.6 mm in height was extracted.^23^ Total area, cortical area, and medullary area were determined from the 2D images using the CT software. The periosteal and endosteal perimeters were calculated using the formula for the area of an ellipse.

### Measurement of tibia length and width

Fresh frozen tibia were evaluated for length and width using digital calipers. For width, the proximal, mid-shaft, and distal regions were averaged. For the length, the distance from growth plate to growth plate was measured.

### Histopathology

Following removal from the Micro-CT scanner, the hind limb bones were frozen at −80 °C until processing. Three processing methods were used to optimally image various bone morphology. A subset of the bones was fixed in formalin with 10% zinc for 24 h, then decalcified for paraffin embedding at the UMMC Histology core for hematoxylin and eosin (H&E) staining and Masson's trichrome. Another subset of bones also was fixed in formalin but was not decalcified and was sent to the Purdue University Core for non-decalcified sectioning and staining with Goldner's trichrome.

### Decalcification of bone

Bones were removed from −20 °C and thawed overnight at 4 °C. The bones then were fixed and decalcified using Cal-EX (Fisher Scientific, Pittsburgh, PA, USA). Once decalcified, sagittal cuts were made. The sagittal-cut bones then were cyroprotected by phase transfer into 300 g/L sucrose in phosphate buffered saline (PBS, pH 7.4). To avoid ice crystal formation, bones were placed in optimal cutting temperature (OCT) compound and flash-frozen using isopentane/2-methylbutane and liquid nitrogen and then stored at −80°C until sectioning. Finally, 10 μM sections were obtained using a cryostat (Leica Biosystems, Buffalo Grove, IL, USA).

### Measurement of the epiphyseal plate

H&E stained slides were scanned using a Philips Slide Digitizer. All images were analyzed at 5 × using internal measurement tools. Each section was measured 3 times at the margins and center of the growth plate, and the averages were calculated and recorded for each group for comparisons.

### Non-decalcified bone processing and staining

Tissue samples were placed in a Leica TP1020 processor for dehydration starting with 70% ethanol, then two changes of 95% ethanol, three changes of 100% ethanol, and two changes of acetone at room temperature for 4 h each. Infiltration was done under vacuum with four changes of 95% methyl methacrylate (Fisher Scientific, 03629-4) and 5% dibutyl phthalate (Fisher Scientific, D30-500) at room temperature for at least 24 h each. Samples were embedded in fresh 95% MMA 5% dibutyl phthalate and polymerized by adding 0.25% Perkadox-16. Tissue sections were taken at a thickness of 5 μm using a Thermo HM355S microtome and tungsten carbide blade. Sections were mounted on charged slides and dried in a metal press overnight in a 60 ℃ oven. After drying, slides for staining were deplasticized through three changes of acetone at room temperature for 10 min each and rehydrated through graded ethanols to water. Stained slides were dehydrated, cleared in xylene, and cover-slipped in a toluene-based mounting media (Leica MM24). Images were taken using a Leica Versa8 whole-slide scanner.

### Statistical analysis

All statistical analyses were performed using GraphPad Prism version 8.1.2 (GraphPad Software, San Diego, CA, USA). Statistical significance was determined with a one-way analysis of variance (ANOVA) followed by Tukey's post hoc test for group comparisons. With multiple regions, we performed two-way ANOVA with drug treatment and region as the variables. All results are given as mean ± standard deviation (SD). Results were considered statistically significant when *p* < 0.05.

## Results

### Changes to ultrastructure and bone mineral density in SCI-VEH, SCI-HU308, and naïve mice

**FIG 1.**
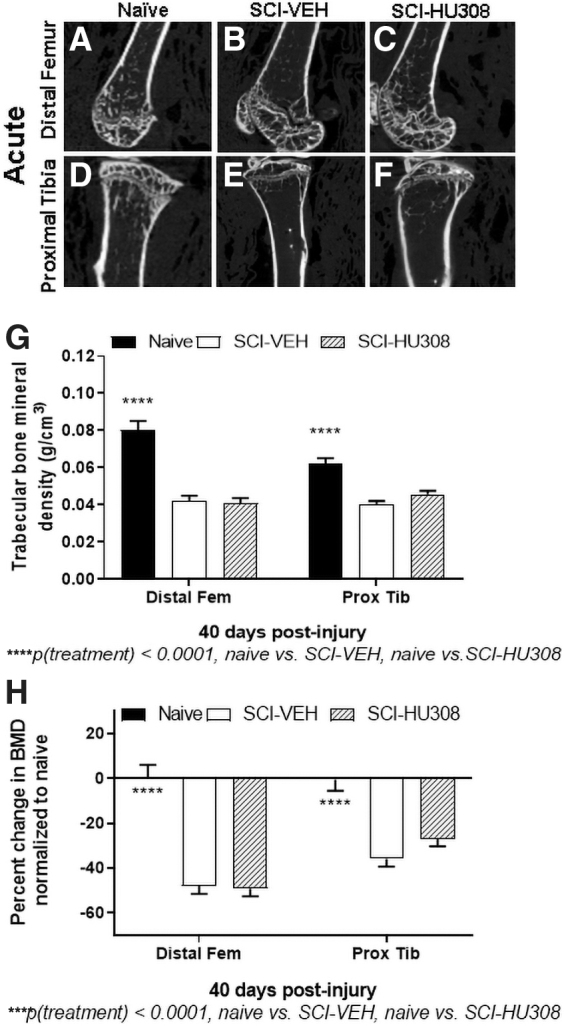
Representative images of distal femurs and proximal tibiae and BMD measurements evaluated after 40 days of acute treatment. 2D Micro-CT cross sections of distal femurs of **(A)** naïve, **(B)** SCI-VEH, and **(C)** SCI-HU308 animals. 2D Micro-CT cross sections of proximal tibiae of **(D)** naïve, **(E)** SCI-VEH, and **(F)** SCI-HU308 animals. (**G**) Trabecular BMD of distal femurs and proximal tibiae. (**H**) Percent difference in BMD normalized to naïve controls. Values reported as mean ± SD. Statistical differences evaluated by one-way ANOVA with Tukey's post hoc test. 2D, two-dimensional; ANOVA, analysis of variance; BMD, bone mineral density; Micro-CT, microtomography; SCI, spinal cord injury; SD, standard deviation; VEH, in vehicle.

In the acute paradigm, gross anatomical measurements of tibia length and width using calipers showed no differences between the groups (data not shown), so Micro-CT reconstructions of tibiae (Fig. 1A–C) and femora (Fig. 1D–F) were performed. Representative images of the distal femur (Fig. 1A–C) and proximal tibia (Fig. 1D–F) are displayed. 3D structural analysis of the metaphyseal region of the distal femur and proximal tibia demonstrated a significant decrease in trabecular BMD in both the SCI-VEH- and SCI-HU308-treated animals compared with naïve controls, *p* < 0.0001 (Fig. 1G). The trabecular BMD in the distal femur was significantly reduced in SCI-VEH by 47.8% and in SCI-HU308-treated animals by 49.1% in comparison to naïve controls (Fig. 1H). We detected an average decrease of 35.9% in the proximal tibia trabecular bone mineral content for SCI-VEH-treated and a reduction of 27.0% for SCI-HU308-treated animals when compared with naïve controls, *p* < 0.0001 (Fig. 1H).

Further Micro-CT measurements were taken of cross sections of the midshaft of distal femurs and tibiae to assess the diaphyseal cortical bone. Total area, cortical area, and periosteal perimeter were not significantly different in both the femurs and tibiae of SCI-VEH- and SCI-HU308-treated animals compared with naïve controls (Table 1). In contrast, the percent of cortical-to-total area was significantly reduced in SCI-VEH animals (*p* < 0.05) and SCI-HU308 animals (*p* < 0.05) by approximately 13% in the femurs compared with naïve control animals (Table 1). The percent of cortical-to-total area in the tibiae also was reduced by 14% in SCI-VEH animals (*p* < 0.001) and by 9% in SCI-HU308 animals (*p* < 0.001) compared with naïve controls (Table 1). The increase in medullary area and endosteal perimeter in SCI-VEH animals and SCI-HU308 animals compared with naïve controls shows the bone marrow canal of the femur is most affected in the acute paradigm, *p* < 0.05 (Table 1).

**Table 1.** Measurements of the Femurs and Tibiae of Mice Evaluated after 40 days of Acute Treatment

	Naïve (A)	SCI-VEH (B)	SCI-HU308 (C)	
Acute treatment	Mean ± SD	Mean ± SD	Mean ± SD	Statistics
Femur				
Total area (mm^2^)	1.74 ± 0.43	2.05 ± 0.46	2.43 ± 0.95	NS
Cortical area (mm^2^)	0.77 ± 0.23	0.73 ± 0.17	0.89 ± 0.38	NS
Medullary area (mm^2^)	0.97 ± 0.20	1.31 ± 0.32	1.54 ± 0.61	A vs. C, *p* < 0.05
Cortical area/Total area (%)	48.88 ± 3.90	35.79 ± 4.75	36.55 ± 5.85	A vs. B, *p* < 0.05, A vs. C, *p* < 0.05
Periosteal perimeter (mm)	4.72 ± 0.52	5.06 ± 0.52	5.52 ± 1.02	NS
Endosteal perimeter (mm)	3.73 ± 4.20	4.30 ± 0.478	4.61 ± 0.78	A vs. C, *p* < 0.05
Tibia				
Total area (mm^2^)	0.95 ± 0.27	1.11 ± 0.37	1.14 ± 0.54	NS
Cortical area (mm^2^)	0.64 ± 0.15	0.60 ± 0.16	0.65 ± 0.28	NS
Medullary area (mm^2^)	0.31 ± 0.13	0.53 ± 0.23	0.49 ± 0.27	NS
Cortical area/Total area (%)	67.65 ± 4.36	53.58 ± 5.08	58.23 ± 5.75	A vs. B, *p* < 0.001, A vs. C, *p* < 0.01
Periosteal perimeter (mm)	3.24 ± 0.471	3.70 ± 0.52	3.78 ± 0.85	NS
Endosteal perimeter (mm)	2.00 ± 0.36	2.61 ± 0.50	2.58 ± 0.64	NS
Growth plate height (μm)	108.6 ± 15.40	85.65 ± 12.97	90.22 ± 19.37	A vs. B, *p* < 0.001, A vs. C, *p* < 0.001

Mice were either injured (SCI) and receiving vehicle (VEH) or CB2 agonist (HU308), or were naïve to both (naïve). Values reported as mean ± SD. Statistical differences evaluated by one-way ANOVA with Tukey's post hoc test.

ANOVA, analysis of variance; NS, not significant; SCI, spinal cord injury; SD, standard deviation; VEH, in vehicle.

**FIG. 2.**
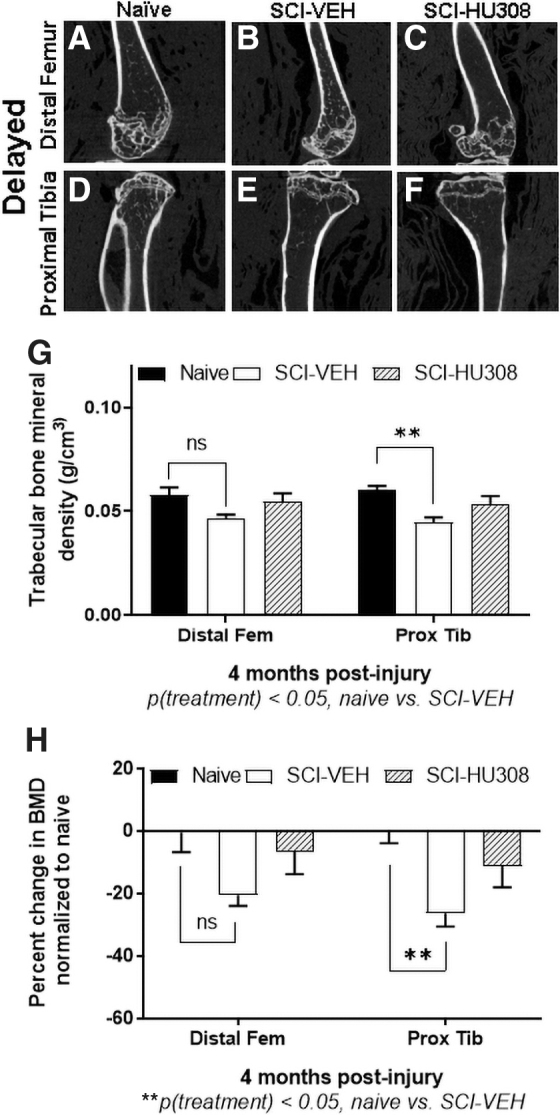
Representative images of distal femurs and proximal tibiae and BMD measurements evaluated at 3 months post-injury plus 30 days of delayed treatment. 2D Micro-CT cross sections of distal femurs of **(A)** naïve, **(B)** SCI-VEH, and **(C)** SCI-HU308 animals. 2D Micro-CT cross sections of proximal tibiae of **(D)** naïve, **(E)** SCI-VEH, and **(F)** SCI-HU308 animals. (**G**) Trabecular BMD of distal femurs and proximal tibiae. (**H**) Percent difference in BMD normalized to naïve controls. Values reported as mean ± SD. Statistical differences evaluated by one-way ANOVA with Tukey's post hoc test. 2D, two-dimensional; ANOVA, analysis of variance; BMD, bone mineral density; Micro-CT, microtomography; SCI, spinal cord injury; SD, standard deviation; VEH, in vehicle.

In the delayed paradigm, the cannabinoid agonist was begun 3 months post-injury and dosed daily for 30 days. Representative Micro-CT reconstructions of the distal femurs and proximal tibiae are displayed using this delayed paradigm (Fig. 2A–F). Measurements obtained from the Micro-CT analysis show that the trabecular bone density was significantly reduced in the SCI-VEH-treated animals in distal femurs and proximal tibiae, when compared with the naïve controls (Fig. 2G) by 20.5% and 26.5%, respectively, *p* < 0.05 (Fig. 2H). SCI animals receiving 10mg/kg HU308 daily showed insignificant reduced loss of trabecular BMD in both distal femurs (6.6%) and proximal tibiae (11.2%), when compared with naïve controls (Fig. 2G,H). HU308-SCI-treated animals showed a significant improvement in trabecular BMD compared with SCI-VEH-treated animals, *p* < 0.05 (Fig. 2G,H). SCI significantly reduced the total area, cortical area, percent of the cortical-to-total area, and both the periosteal and endosteal perimeters (Table 2) in femurs. Treatment with HU308 in animals produced an increased total area, cortical area, and endosteal perimeter when compared with SCI-VEH treatment in animals, *p* < 0.01 (Table 2), suggesting HU308 may have more of an effect on the periosteal bone. Tibial cortical bone parameters did not differ between the treated groups, but were significantly lower than in the naïve controls, *p* < 0.01 (Table 2).

**Table 2.** Measurements of the Femurs and Tibiae of Mice Evaluated at 3 Months Post-Injury plus 30 Days of Delayed Treatment

	Naïve (A)	SCI-VEH (B)	SCI-HU308 (C)	
Delayed treatment	Mean ± SD	Mean ± SD	Mean ± SD	Statistics
Femur				
Total area (mm^2^)	2.12 ± 0.19	1.69 ± 0.20	1.81 ± 0.154	A vs. B, *p* < 0.001, A vs. C, *p* < 0.01
Cortical area (mm^2^)	0.92 ± 0.11	0.63 ± 0.11	0.72 ± 0.08	A vs. B, *p* < 0.001, A vs. C, *p* < 0.01
Medullary area (mm^2^)	1.19 ± 0.18	1.06 ± 0.12	1.08 ± 0.11	NS
Cortical area/Total area (%)	43.6 ± 5.16	36.98 ± 3.56	40.22 ± 3.13	A vs. B, *p* < 0.05
Periosteal perimeter (mm)	5.18 ± 0.36	4.68 ± 0.39	4.81 ± 0.28	A vs. B, *p* < 0.05
Endosteal perimeter (mm)	4.24 ± 0.37	3.72 ± 0.17	3.69 ± 0.24	A vs. B, *p* < 0.01, A vs. C, *p* < 0.01
Tibia				
Total area (mm^2^)	1.19 ± 0.15	0.91 ± 0.139	0.928 ± 0.16	A vs. B, *p* < 0.01, A vs. C, *p* < 0.01
Cortical area (mm^2^)	0.73 ± 0.12	0.49 ± 0.08	0.54 ± 0.14	A vs. B, *p* < 0.01, A vs. C, *p* < 0.01
Medullary area (mm^2^)	0.46 ± 0.10	0.42 ± 0.97	0.39 ± 0.06	NS
Cortical area/Total area (%)	61.39 ± 6.17	53.96 ± 6.11	57.46 ± 6.79	NS
Periosteal perimeter (mm)	3.82 ± 0.49	3.27 ± 0.23	3.37 ± 0.23	A vs. B, *p* < 0.05, A vs. C, *p* < 0.05
Endosteal perimeter (mm)	2.16 ± 0.24	2.29 ± 0.26	2.13 ± 0.30	NS
Growth plate height (μm)	111.2 ± 19.12	78.38 ± 16.99	89.9 ± 16.25	A vs. B, *p* < 0.001, A vs. C, *p* < 0.001 and B vs. C, *p* < 0.01

Mice were either injured (SCI) and receiving vehicle (VEH) or CB2 agonist (HU308), or were naïve to both (naïve). Values reported as mean ± SD. Statistical differences evaluated by one-way ANOVA with Tukey's post hoc test.

ANOVA, analysis of variance; NS, not significant; SCI, spinal cord injury; SD, standard deviation; VEH, in vehicle.

### Bone histological assessment following treatment of SCI with HU308

**FIG. 3.**
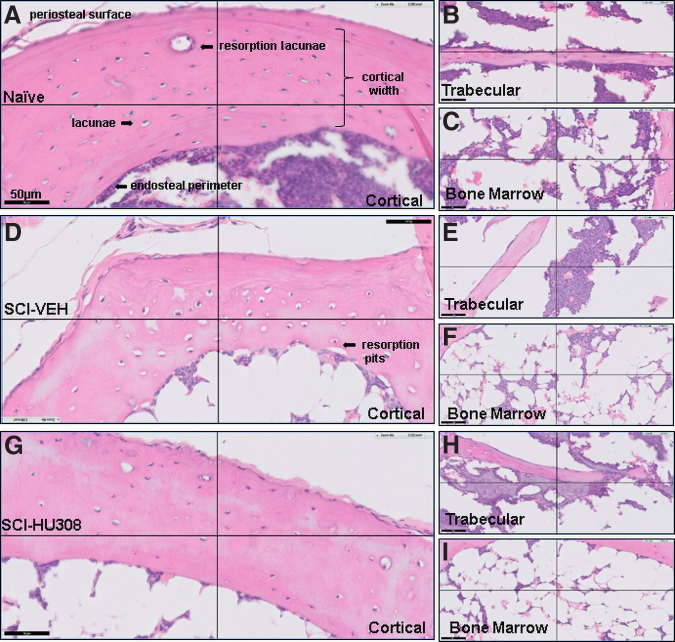
Histological staining of decalcified proximal tibiae from mice evaluated after 40 days of acute treatment. Photomicrograph of H&E staining of cortical bone in (**A**) naïve, (**D**) SCI-VEH, and (**G**) SCI-HU308 animals. Photomicrograph of H&E staining of trabecular bone in (**B**) naïve, (**E**) SCI-VEH, and (**H**) SCI-HU308 animals. Photomicrograph of H&E staining of bone marrow in (**C**) naïve, (**F**) SCI-VEH, and (**I**) SCI-HU308 animals. Images obtained using a Philips Slide Digitizer. Bar represents 50 μm. Periosteal surface and resorption pits in cortical region are labeled. H&E, hematoxylin and eosin; SCI, spinal cord injury; VEH, in vehicle.

**FIG. 4.**
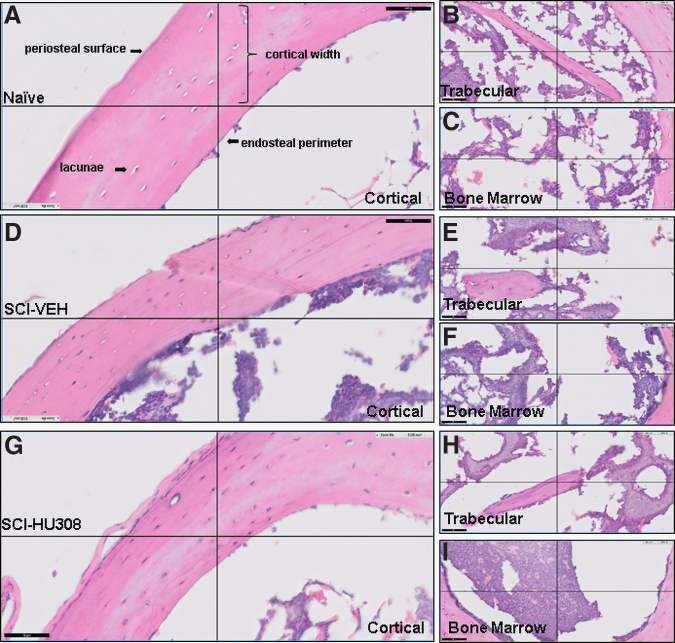
Representative histological staining of decalcified proximal tibiae from mice evaluated at 3 months post-injury plus 30 days of delayed treatment. Photomicrograph of H&E staining of cortical bone in (**A**) naïve, (**D**) SCI-VEH, and (**G**) SCI-HU308 animals. Photomicrograph of H&E staining of trabecular bone in (**B**) naïve, (**E**) SCI-VEH, and (**H**) SCI-HU308 animals. Photomicrograph of H&E staining of bone marrow in (**C**) naïve, (**F**) SCI-VEH, and (**I**) SCI-HU308 animals. Images obtained using a Philips Slide Digitizer. Bar represents 50 μm. Periosteal surface and resorption pits in cortical region are labeled. H&E, hematoxylin and eosin; SCI, spinal cord injury; VEH, in vehicle.

H&E-stained slides were used to qualitatively evaluate and compare trabecular bone in the tibia metaphysis and cortical bone in the diaphysis for comparison of bone thinning, location, number, and morphology of osteocytes and osteoclasts (Tables 3 and 4, Fig. 3A–I, and Fig. 4A–I). In the acute paradigm, the cortical bone width was larger in naïve controls than in both SCI-VEH- and SCI-HU308-treated animals (Fig. 3A,D,G). The number of osteocytes per unit area was not statistically different in diaphyseal region of cortical bone between the treatment groups and naïve controls (Table 3); however, the shape of the osteocytes were distinctly different from that in naïve controls. The osteocytes in bone in naïve controls appeared flattened compared with those seen in SCI-VEH-treated and SCI-HU308-treated animals. The osteocytes in the SCI-VEH-treated animals had larger lacunae than in naïve controls or SCI-HU308-treated animals. The number of osteoclasts in the cortical bone was significantly reduced in the SCI-HU308-treated animals compared with SCI-VEH and naïve animals. It is also interesting to note the changes on the endocortical side of the bone. In the naïve animals, there is a clear presence of a flattened and continuous bone cell lining. In both SCI-VEH- and SCI-HU308-treated animals, the bone cells were more rounded and did not form continuous lining. Naïve control animals had an appearance of more robust cell populations in the marrow cavity compared with either treated group (Fig. 3C,F,I).

**Table 3.** Number of Osteocytes and Osteoclasts in Diaphyseal and Metaphyseal Region of the Tibiae from Mice after 40 Days of Acute Treatment, or 3 Months Post-Injury plus 30 Days of Delayed Treatment

Proximal tibia cell counts	Naïve (A)	SCI-VEH (B)	SCI-HU308 (C)	
(per unit area)	Mean ± SD	Mean ± SD	Mean ± SD	Statistics
Acute				
Diaphyseal osteocytes	26.04 ± 13.09	25.33 ± 10.44	28.28 ± 9.09	NS
Diaphyseal osteoclasts	26.37 ± 11.17	29.61 ± 11.44	18.9 ± 10.42	B vs. C, *p* < 0.05
Metaphyseal osteocytes	37.02 ± 58.49	87.94 ± 58.49	35.82 ± 12.52	A vs. B, *p* < 0.05, A vs. C, *p* < 0.05
Metaphyseal osteoclasts	23.80 ± 9.44	52.00 ± 37.9	29.65 ± 13.30	A vs. B, *p* < 0.05, A vs. C, *p* < 0.05
Delayed				
Diaphyseal osteocytes	38.00 ± 17.19	31.14 ± 11.07	36.14 ± 10.90	NS
Diaphyseal osteoclasts	18.47 ± 8.56	18.87 ± 5.93	13.81 ± 5.55	NS
Metaphyseal osteocytes	54.98 ± 27.67	43.73 ± 12.00	39.16 ± 11.11	NS
Metaphyseal osteoclasts	22.88 ± 7.18	21.19 ± 11.42	24.80 ± 13.67	NS

Data are expressed as mean cell number/μm ± SD. Asterisks designates significances (*n* = 3 samples per group with 4 averaged measurements). Values reported as mean ± SD.

NS, not significant; SCI, spinal cord injury; SD, standard deviation; VEH, in vehicle.

**Table 4.** Measurements of the Region of Interest of the Proximal Tibiae from Mice after 40 Days of Acute Treatment, or 3 Months Post-Injury plus 30 Days of Delayed Treatment

	Naïve (A)	SCI-VEH (B)	SCI-HU308 (C)	
	Mean ± SD	Mean ± SD	Mean ± SD	Statistics
Acute				
Bone volume fraction (%)	12.93 ± 3.23	13.22 ± 4.36	12.26 ± 1.75	NS
Trabecular thickness (Tb.Th) (mm)	0.13 ± 0.01	0.12 ± 0.01	0.16 ± 0.04	NS
Trabecular separation (Tb.Sp) (mm)	0.46 ± 0.07	0.71 ± 0.18	0.76 ± 0.22	A vs. B, *p* < 0.05, A vs. C, *p* < 0.05
Trabecular number (Tb.N) #/mm	1.15 ± 0.01	0.98 ± 0.43	0.62 ± 0.52	NS
Delayed				
Bone volume fraction (%)	11.70 ± 2.82	8.57 ± 0.39	8.92 ± 0.46	NS
Trabecular thickness (Tb.Th) (mm)	0.17 ± 0.02	0.14 ± 0.04	0.13 ± 0.03	NS
Trabecular separation (Tb.Sp) (mm)	0.44 ± 0.02	0.53 ± 0.27	0.62 ± 0.37	NS
Trabecular number (Tb.N) #/mm	0.96 ± 0.14	0.75 ± 0.08	0.77 ± 0.21	NS

Values reported as mean ± SD. Statistical differences evaluated by one-way ANOVA with Tukey's post hoc test; *n* = 3/group.

ANOVA, analysis of variance; NS, not significant; SCI, spinal cord injury; SD, standard deviation; VEH, in vehicle.

It is also important to note that the lack of a continuous lining of bone may be due to changes in mechanical sensing of load and disrupted cellular communication as evidenced by the shape change of the osteocytes. Additional changes also were noted in the SCI-VEH-treated animals, such as the presence of resorption pits that were evident on the endocortical side (Fig. 3D), compared with both naïve controls (Fig. 3A) and SCI-HU308-treated animals (Fig. 3G). Lack of visible resorption pits on the endocortical side along with the decrease in osteoclast numbers in the HU308-treated animals lends further support to the possible inhibition of osteoclastogenesis via CB2 receptor agonism.

Differences also were noted in metaphyseal region of the highly metabolic trabecular bone in the proximal tibiae between the groups. There were significantly increased numbers of osteocytes and osteoclasts in the SCI-VEH-treated animals in the acute paradigm compared with naïve controls and SCI-HU308-treated animals (Table 3) along with the appearance of spicules. The trabecular bone in the naïve control animals appeared longer and thinner with more bone-lining cells when compared with the SCI-VEH and SCI-HU308 animals (Fig. 3 B,E,H). Scanning a region of interest encompassing approximately 0.85 mm of the proximal tibia metaphysis adjacent to the growth plate showed no significant differences in bone volume, but did show a trend for structural changes as the trabecular separation in both SCI-VEH and SCH-HU308 animals was approximately 40% greater than that seen in naïve animals (Table 4).

In the delayed paradigm, naïve controls had thicker cortical bone (Fig. 4A,D,G). Interestingly, the number of osteocytes in the cortical bone appeared greater in the SCI animals when compared with the naïve animals; however, when counted and normalized to area, the osteocyte numbers were not statistically different (Table 3). In evaluating the number of osteoclast cells, SCI-HU308-treated animals had lower numbers of osteoclasts on average compared with SCI-VEH and naïve animals, but these were not statistically different (Table 3). Cement lines were visible in the SCI-VEH-treated animals, which indicate active bone remodeling. The bone marrow of the SCI-VEH- and SCI-HU308-treated animals appeared more cellular than in naïve controls (Fig. 4C,F,I). The SCI-HU308-treated animals had nodules of mineralized bone in the marrow cavity (Fig. 4I). Analysis of the metaphyseal trabecular bone in the delayed paradigm shows a trend toward a decrease in bone volume and trabecular number along with increased trabecular separation in both SCI-VEH and SCI-HU308 animals. Histological assessment of the trabecular bone revealed basic multi-cellular units (BMU) located on the bone surface and covered by a thin canopy of elongated mesenchymal cells covering the whole bone remodeling area and separating it from the bone marrow (Fig. 4B,E,H). This can be seen more readily in both the naïve controls and SCI-HU308 animals in the delayed paradigm compared with SCI-VEH animal trabeculae.

### Growth plate changes following SCI and treatment with HU308

**FIG. 5.**
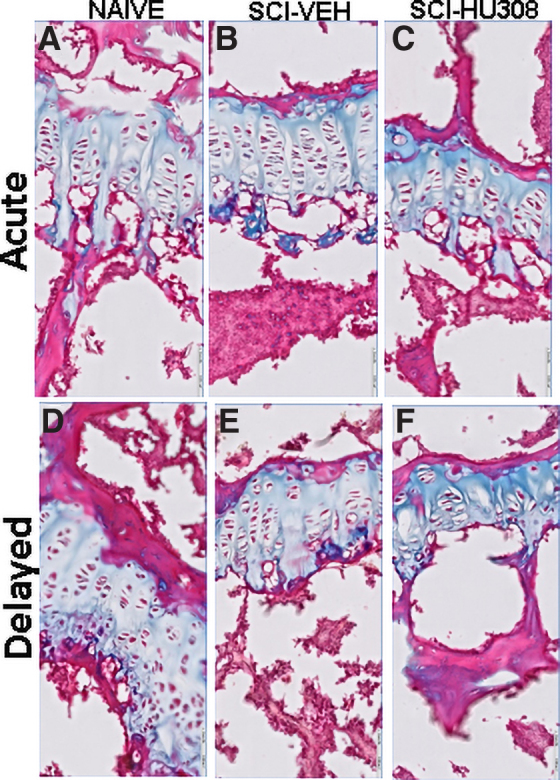
Representative histological staining of the growth plate from decalcified proximal tibiae in both treatment paradigms. Photomicrographs of Goldner's stain from acute treatment (**A**) naïve, (**B**) SCI-VEH, and (**C**) SCI-HU308 animals, and delayed treatment (**D**) naïve, (**E**) SCI-VEH, and (**F**) SCI-HU308 animals. Bar represents 50 μm. SCI, spinal cord injury; VEH, in vehicle.

Digital measurements of the height of the proximal tibia growth plate within each group were fairly uniform. In the acute paradigm, there was a significant decrease in the height of the growth plate, *p* < 0.001, in SCI-VEH-treated animals (21%) and SCI-HU308-treated animals (16.7%) compared with naïve controls (Table 1). In the delayed paradigm, the growth plate height in SCI-VEH and SCI-HU308 animals also was significantly reduced in comparison to naïve controls; however, some benefit was realized in that the growth plate was increased with CB2 agonist treatment, *p* < 0.01 (Table 2). In addition, several features were found in the SCI-injured animals that were not present in the naïve controls. In the acute paradigm, the SCI-VEH-treated animals had wider columns compared with both the naïve controls and SCI-HU308-treated animals (Fig. 5A–C). In the delayed paradigm, the boundaries between the proliferating and hypertrophic zones were less distinct along with a reduction in chondrocyte numbers in the SCI-VEH-treated animals (Fig. 5D–F). SCI-HU308-treated animals had substantially fewer chondrocytes in the hypertrophic and proliferating zones as well as columns completely devoid of cells (Fig. 5D–F).

### Metaphyseal and diaphyseal periosteum changes using Goldner's stain following SCI and treatment with HU308

**FIG. 6.**
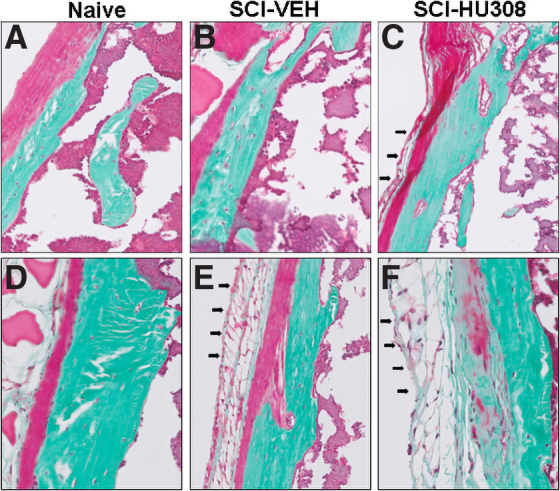
Representative histological staining of the periosteum from decalcified proximal tibiae in both treatment paradigms. Photomicrographs of Goldner's stain from acute treatment (**A**) naïve, (**B**) SCI-VEH, and (**C**) SCI-HU308 animals, and delayed treatment (**D**) naïve, (**E**) SCI-VEH, and (**F**) SCI-HU308 animals. Bar represents 50 μm. Arrows illustrate the fat deposition. SCI, spinal cord injury; VEH, in vehicle.

The metaphyseal periosteum appeared thicker and more fibrous than the diaphyseal periosteum in all animals in both the acute and delayed paradigm (Fig. 6). The fibrous band in the metaphyseal periosteum was thinner with a more cellular cambial layer in the SCI animals compared with naïve controls (Fig. 6B). An increase in adipocytes, which were loosely grouped together in lobules and separated by connective tissue, comprised the last layer of the periosteum in SCI-HU308-treated animals (Fig. 6C), which was not evident in the SCI-VEH or naïve animals (Fig. 6AB).

In the delayed paradigm, the fibrous tissue layer in the metaphyseal periosteum of the SCI-VEH-treated animals was thinner and less cellular than the metaphyseal periosteum of naïve controls (Fig. 6D–F). In addition, there was an increase in the number of adipocytes, increased thickness of the final fatty layer of the periosteum in SCI-VEH animals, and an absence of a fatty layer in naïve animals (Fig. 6D,E). The fibrous bands in the metaphyseal periosteum in SCI-H308-treated animals were replaced by fibrocartilage with osteoid (Fig. 6F). The tissue adjacent to this region consisted of loose fatty connective tissue (Fig. 6F). The staining demonstrated collagen production in the fat layer (Fig. 6F).

## Discussion

Bone undergoes constant remodeling, which requires coordinated osteocyte, osteoblast, and osteoclast cell activities for optimal bone homeostasis. After SCI in humans, disruption of the activities of the various cell types favors bone resorption, leading to severe bone loss, particularly in the sublesional appendicular bones; this can lead to an increased risk of low-impact fractures. In this study, we used a spinal transection lesion at T8 in adult mice and performed a comprehensive analysis of bone structure following the administration of a CB2 agonist, HU308. HU308 has been shown to effectively improve menopausal bone loss by interfering with osteoclast development and activity.^24,25^ HU308 was administered daily at either the onset of injury or after osteoporosis was established.

### Acute CB2 agonism does not preserve bone loss

In the acute phase, administration of the CB2 agonist was not able to preserve the rapid loss of cancellous bone, which differs from rodent models of ovariectomy-induced osteoporosis where significant bone preservation was seen after 19 days of continuous administration of a CB2 agonist.^24,25^ MicroCT analysis of the proximal tibiae and distal femurs showed SCI causes a significant reduction in trabecular bone density with trabecular separation. Also, our data show site-specific changes in osteocyte and osteoclast numbers in the metaphyseal region compared with the diaphyseal region in SCI animals. Significantly more osteocytes per bone tissue volume were measured in the metaphyseal region in SCI-VEH-treated animals in the acute phase when compared with both naïve controls and SCI-HU308-treated animals.

The increase in osteocyte density has also been reported in human osteoporosis studies^26^ as well as in studies related to fracture healing.^27^ In a study of bone turnover after SCI, pro-inflammatory markers were elevated in osteocytes, and the authors concluded that osteocyte inflammation is a contributor to the significant bone loss in a rodent model of SCI.^28^ In the diaphyseal region, no differences were seen in osteocyte or osteoclast numbers per unit area between the groups. However, the morphology of the osteocytes in this region differed in shape. The osteocytes in naïve controls and SCI-HU308 animals appeared flattened without a large perinuclear halo when compared with SCI-VEH animals. In various pathological and physiological conditions such as lactation, immobilization of bone, and hyperparathyroidism, the osteocytes are capable of demineralizing the perilacunar matrix to maintain calcium homeostasis and hence, contribute to bone resorption.^29^ Currently, there is little information in the literature on osteocyte-induced bone resorption and how it differs from osteoclastic bone resorption in SCI. The anti-inflammatory properties associated with the CB2 agonist, HU308, may contribute to these changes.^24,30,31^ More investigation is needed to determine the role of osteocyte inflammatory markers in relation to degradation of the lacunae and the role CB2 agonism plays in preventing osteocyte inflammation.

### Acute CB2 agonism does not suppress SCI-induced changes to the growth plate

In addition to bone loss, we also observed growth plate and periosteum changes in the acute phase. In the growth plate, we noted a loss of normal cellular organization with areas of acellularity, and on the other hand, hypercellularity in conjunction with metaphyseal bone loss. Growth plate abnormalities have been reported in rodent SCI models.^32^ It is interesting to note in the hyperproliferative zones there is evidence of asymmetric cell division with two cells inside the same lacunae. The changes in the symmetry of the growth plate may contribute to the flattened appearance of the tibial plateau. Flattening of the tibial plateau can result in spontaneous collapse and increased fracture risk.^33–35^ Early administration of HU308 was unable to suppress the growth plate changes.

### Acute CB2 agonism produces periosteal changes in SCI-induces osteoporosis

In our study, we also found structural and cellular changes in the metaphyseal periosteum. We identified increased cells in the cambial layer and thicker fibrous tissue in the acute phase. Fan and colleagues^36^ reported structural changes in periosteum of osteoporotic rats.^36^ They also found thicker fibrous area and increased cellular cambial layer in the metaphyseal region.^36^ However, we are the first to report increased presence of adipocyte and fat mass in the periosteum of SCI animals, which was not present in the naïve controls. The layer of fat increased markedly in SCI-HU308-treated animals compared with SCI-VEH animals. The cells in the cambial layer of the periosteum are highly proliferative and osteogenic in response to mechanical stimulation. Because mesenchymal cells also are present in the periosteum, they can differentiate into chondroblast and adipocytes.^37^

A reciprocal relationship has been demonstrated between adipocyte and osteoblast differentiation in the bone marrow with adipocyte differentiation primarily controlled by peroxisome proliferator receptor ɣ (PPARɣ) during skeletal unloading.^38^ The deposition of fat in the periosteal layer also may control bone remodeling. Han and associates, using a hindlimb unloading rodent model, found muscle atrophy and downregulation of insulin-like growth factor-1 (IGF-1).^39^ The periosteal surface also is responsive to IGF-1 and is important for parathyroid-driven periosteal apposition.^40^ In unloaded bone, there is a dysregulation of the calcium/parathyroid hormone loop^41^ along with disruption of tissue IGF-1 axis, which also may contribute to changes in bone remodeling and bone quality.

### Improvements observed in delayed treatment with CB2 agonism

In our study we found that in the chronic phase osteocytes and osteoclasts were similar per unit area to that in naïve controls in both the metaphyseal and diaphyseal area. In both the SCI-VEH- and SCI-HU308-treated animals, there was an improvement of cellularity of the bone marrow in the delayed paradigm. The bone marrow of SCI-HU308-treated animals showed an increased presence of osteoblast and bone nodules, which were not as evident in SCI-VEH animals. Disorganization and asymmetry of the growth plate still was observed in the SCI-VEH and SCI-HU308 animals compared with naïve animals; however, there was greater evidence of chondrocyte hypertrophy and bone spicules seen at the edge of the growth plate in SCI-HU308-treated animals compared with SCI-VEH and naïve animals.

Roach and colleagues found asymmetric cell division in hypertrophic chondrocytes of the growth plate in chicks and aging rodents resulting in transdifferentiation of the chondrocytes to bone-forming cells.^44,45^ The periosteum in SCI animals had significant increases in adipocytes when compared with naïve animals. Adipocytes are both mechano-sensing and mechano-responsive.^44,45^ Studies on the effect of mechanical forces loaded on stem cells, pre-adipocytes, and adipocytes result in totally different effects on adipogenesis. In general, static stretch promotes adipogenesis, whereas, cyclic stretching and static compression inhibit adipogenesis.^45^ Therefore, the lack of cyclic stretching following SCI contributes to the increased presence of adipocytes in the periosteum.

It is interesting to note that cannabinoids are capable of activating PPARɣ,^46^ which may potentiate the increase in adipocyte differentiation in the periosteum. It also is worth mentioning that mechano-transductive signals are important for tissue regeneration. In bone, the effects of fluid flow and loading are important determinants for osteocyte-mediated bone formation. Mechanical forces cause direct stretching of protein-cell surface integrin-binding sites that occur on all eukaryotic cells. The loss of the fibrous tissue in the periosteal layer alters how the osteocytes perceive signals because stress-induced conformational changes in the extracellular matrix may alter integrin structure and lead to activation of several secondary messenger pathways within the cell. Activation of these pathways leads to altered regulation of genes that synthesize and catabolize extracellular matrix. It cannot be ruled out that in the chronic phase, the modest increase in trabecular bone spicules and bone nodules in the marrow cavity seen in SCI-HU308-treated animals is related to multi-factorial processes that include the proliferation of adipocytes in the periosteum providing mechanotransduction that triggers the activation of ion channels,^47^ which increase calcium permeability and ion fluxes that lead to the activation of the osteocytes to coordinates bone marrow differentiation of osteoblasts. Additional studies are needed to address the changes in the periosteum and how they affect the quality of bone strength in the chronic phase following HU308 treatment.

## Conclusion

Chronic SCI and subsequent osteoporosis have a significant impact on an individual's health and places an economic burden on the individual and the healthcare system. Currently, there is no therapeutic treatment for SCI-induced osteoporosis in the chronic phase. Bone loss following SCI far exceeds that seen in menopausal and mechanical unloading. Therefore, there is a significant need to produce effective treatment strategies to improve care for this vulnerable population. Treatment with a CB2 agonist, HU308, is most effective when used in the delayed paradigm (mimicking chronic SCI) we outline here. However, future work should define the dose and kinetics of bone loss with relationship to bone markers when mechanical unloading and most bone loss occurs. In this study, we utilized only one dose in both the acute and delayed paradigm. It may be possible that the accelerated bone loss in the acute phase would respond more effectively to a higher dose of HU308. Further, we dosed the animals using daily doses rather than continuous administration through a pellet or mini-pump. These altered dosing strategies may have improved the outcomes for both acute and delayed treatments while reducing stress associated with injection and possibly an overall reduction in the concentration of the drug used.

We also report noticeable changes in the metaphyseal periosteum following SCI and treatment with HU308; these have never been reported to our knowledge and may have a meaningful impact on periosteal bone apposition in chronic SCI. Further, the changes in the metaphyseal periosteum along with the disorganized growth plate in SCI are very pertinent to pediatric SCI where longitudinal growth is disrupted dramatically. Understanding the dynamics of the growth plate in development may improve bone quality and stature in this vulnerable population.

It was not in the current scope of the present study to measure hormonal or bone biomarkers in plasma or urine; these would be interesting to obtain in future studies. The collection of bone marrow aspirates would also expand our understanding of the osteocyte communication with mesenchymal and hematopoietic systems.

## Abbreviations Used

2D: two-dimensional
3D: three-dimensional
ANOVA: analysis of variance
BMD: bone mineral density
BMU: basic multi-cellular units
CB1: cannabinoid 1
CB2: cannabinoid 2
CT: computed tomography
H&E: hematoxylin and eosin
IGF-1: insulin-like growth factor-1
IP: intraperitoneal
Micro-CT: microtomography
OCT: optimal cutting temperature
PBS: phosphate buffered saline
PPARɣ: peroxisome proliferator receptor ɣ
SCI: spinal cord injury
SD: standard deviation
T8: thoracic vertebral level 8
UMMC: University of Mississippi Medical Center
VEH: in vehicle
VOI: volume of interest
